# Biphasic Effect of Curcumin on Morphine Tolerance: A Preliminary Evidence from Cytokine/Chemokine Protein Array Analysis

**DOI:** 10.1093/ecam/neq018

**Published:** 2011-06-05

**Authors:** Jui-An Lin, Jenn-Han Chen, Yuan-Wen Lee, Chao-Shun Lin, Ming-Hui Hsieh, Chuen-Chau Chang, Chih-Shung Wong, Judy Ju-Yi Chen, Geng-Chang Yeh, Feng-Yen Lin, Ta-Liang Chen

**Affiliations:** ^1^Graduate Institute of Clinical Medicine, College of Medicine, Taipei Medical University, Taiwan; ^2^Department of Anesthesiology, Taipei Medical University Hospital, Taipei Medical University, Taiwan; ^3^Department of Anesthesiology, School of Medicine, College of Medicine, Taipei Medical University, Taiwan; ^4^Department of Anesthesiology, Anesthetics and Toxicology Research Center, Taipei Medical University Hospital, Taipei Medical University, Taiwan; ^5^Cancer Center, Wan-Fang Hospital, Taipei Medical University, Taiwan; ^6^Department of Anesthesiology, Cathay General Hospital, Taipei, Taiwan; ^7^Pritzker School of Medicine, University of Chicago, IL, USA; ^8^Department of Pediatrics, Taipei Medical University Hospital, Taipei, Taiwan

## Abstract

The aim of this study was to evaluate the effect of curcumin on morphine tolerance and the corresponding cytokine/chemokine changes. Male ICR mice were made tolerant to morphine by daily subcutaneous injection for 7 days. Intraperitoneal injections of vehicle, low-dose or high-dose curcumin were administered 15 min after morphine injection, either acutely or chronically for 7 days to test the effect of curcumin on morphine-induced antinociception and development of morphine tolerance. On day 8, cumulative dose-response curves were generated and the 50% of maximal analgesic dose values were calculated and compared among groups. Corresponding set of mice were used for analyzing the cytokine responses by antibody-based cytokine protein array. Acute, high-dose curcumin enhanced morphine-induced antinociception. While morphine tolerance was attenuated by administration of low-dose curcumin following morphine injections for 7 days, it was aggravated by chronic high-dose curcumin following morphine injection, suggesting a biphasic effect of curcumin on morphine-induced tolerance. Of the 96 cytokine/chemokines analyzed by mouse cytokine protein array, 14 cytokines exhibited significant changes after the different 7-day treatments. Mechanisms for the modulatory effects of low-dose and high-dose curcumin on morphine tolerance were discussed. Even though curcumin itself is a neuroprotectant and low doses of the compound serve to attenuate morphine tolerance, high-doses of curcumin might cause neurotoxicity and aggravate morphine tolerance by inhibiting the expression of antiapoptotic cytokines and neuroprotective factors. Our results indicate that the effect of curcumin on morphine tolerance may be biphasic, and therefore curcumin should be used cautiously.

## 1. Introduction

While opioids are the drug of choice for the alleviation of acute and chronic pain, opioid tolerance, which refers to the diminution of the analgesic effect or the need for a higher dose to maintain the original effect after chronic opioid exposure, remains a perpetual concern, especially since the mechanisms underlying the development of tolerance are complex and still unclear [1]. Recent studies indicate that repeated morphine exposure progressively activates the spinal cord glia, resident immune cells of the central nervous system (CNS), resulting in the release of proinflammatory cytokines that trigger nitric oxide and p38 mitogen-activated protein kinase and lead to the blocking of morphine-induced antinociception in tolerant subjects [2]. Gene therapy for the release of anti-inflammatory cytokines also potentiates acute, morphine-induced antinociception and attenuates the development of morphine tolerance [3]. Furthermore, the use of neutralizing antibodies against chemokine receptors modulates the antinociceptive effect of morphine and reduces morphine tolerance [3]. These observations suggest that the actions of cytokines/chemokines could play a key role in the development of morphine tolerance.

Curcumin (diferuloylmethane) is a yellow-colored phenolic pigment, the active constituent of *Curcuma longa*, and is extensively used as a spice as well as a food preservation and coloring material in India, China and Southeast Asia [4]. Several therapeutic effects of curcumin have been reported [5], including the ability to attenuate diabetic neuropathic pain through a dose-dependent inhibition of the release of proinflammatory cytokines [6, 7]. While neuropathic pain and morphine tolerance are two seemingly unrelated phenomena, they share a common central neuroplastic pathway [8]. Furthermore, drugs for neuropathic pain and opioid tolerance are possibly interchangeable in some aspects and the biochemical alterations observed in opioid tolerance might also be found in neuropathic pain [9]. An oral low-dose curcumin treatment of 10−100 mg kg^−1^ taken 1 h before morphine administration once daily for 5 consecutive days resulted in a dose-dependent reduction of morphine tolerance and the disappearance of the upregulation of brain-derived neurotrophic factor, an anti-opioid substance [10]. However, the effect of high-dose curcumin and its interaction with cytokine/chemokine production have not yet been examined with regard to morphine tolerance. Another report showed that the intraperitoneal (i.p.) administration of curcumin produced a dose-dependent inhibition of facial grooming in both acute and tonic phases in rats with formalin-induced orofacial pain, and high-dose curcumin (400 and 600 mg kg^−1^) exhibited the greatest suppressive effect [11]. Using this dosage, we investigated whether high-dose curcumin also attenuates morphine tolerance in the same dose-dependent manner. The excellent tolerance of curcumin as a food additive with minimal side effects was observed when high doses were taken by healthy volunteers [12]. However, the typical expression of hormesis, the biphasic dose response, of curcumin has been reported [13]. Furthermore, some of the effects of curcumin at high doses *in vitro* are clearly toxic and undesirable beyond its use in cancer therapy [14]. Therefore, the purpose of this investigation was to evaluate the dose effects of curcumin on morphine tolerance and the corresponding cytokine/chemokine responses.

## 2. Methods

### 2.1. Animals

Male ICR mice in the weight range of 18−22 g were used in all experiments. The mice were housed in a room with a 12-h light/dark cycle and given free access to a laboratory standard-fat diet and tap water. The use of animals in this study conformed to the Guiding Principles in the Care and Use of Animals as approved by the Council of the American Physiology Society and by the Taipei Medical University.

### 2.2. Drugs and Injection Methods

**Figure 1:**
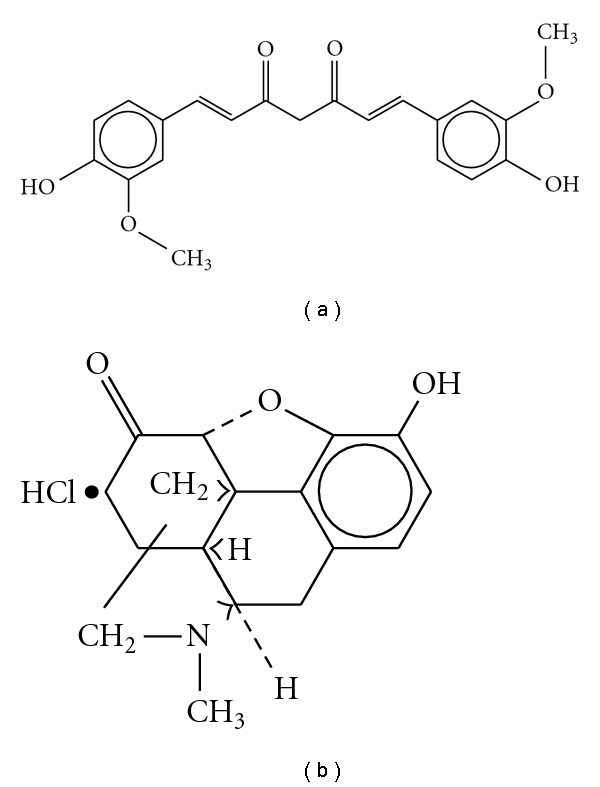
Chemical structures of curcumin (a) and morphine hydrochloride (b).

All of the mice received 100 *μ*L injections. Morphine hydrochloride (morphine-HCl) was dissolved in physiological saline and injected subcutaneously (s.c.), whereas curcumin (Sigma Co., St Louis, Missouri, USA) was dissolved in 70% dimethyl sulfoxide (DMSO) and injected i.p. The chemical structures of morphine and curcumin are shown in Figure 1. On the basis of the cumulative evidences of discrepancies in the effects of curcumin, we chose the i.p. route for its administration. Since DMSO can alter morphine antinociception after both acute (enhancement) and chronic (inhibition) administrations depending on its concentration [15], we also examined the effect of DMSO on morphine antinociception using the following study design.

### 2.3. Antinociceptive Test, Induction of Morphine Tolerance and Body Weight Measurement

Tail-flick latency in the hot-water immersion test (52°C ± 0.1°C) was measured to assess the antinociceptive effect and the development of tolerance in mice receiving a morphine injection either alone or in combination with a low or high dose of curcumin. Before each test, the mice were placed in a plastic restrainer for 30 min to acclimatize. A maximum hot water exposure (cut-off) time of 10 s was used to avoid tissue damage. Previous studies have shown that morphine-HCl (10 mg kg^−1^, s.c.) exhibits its maximal analgesic effect—from 15 to 60 min after administration [10] and plasma concentrations of curcumin reach their maximum concentrations 15 min after i.p. administration [16]. Therefore, the mice were first treated with morphine-HCl (10 mg kg^−1^, s.c.), then treated with DMSO or curcumin (25 or 400 mg kg^−1^, i.p.) and evaluated in the tail-flick test 15 min after injection of DMSO or curcumin. To study the acute effects of DMSO and curcumin (25 or 400 mg kg^−1^, i.p.) on the antinociceptive effect of morphine (1 mg kg^−1^, s.c.), tail-flick tests were conducted every 15 min after the final drug injection for a 90-min period. In experiments exploring the effect of chronic curcumin treatment on the development of morphine tolerance, mice were given morphine-HCl (10 mg kg^−1^, s.c.) followed 15 min later by DMSO (100 *μ*L, i.p.) or curcumin (25 or 400 mg kg^−1^, i.p.) daily for 7 days. To determine the cumulative dose-response curve on Day 8, tail-flick latencies were converted into maximum percent effect (MPE) using the equation:

(1) $$\begin{array}{cc} & \text{MPE}\;(\%)\\ & =\;\frac{\text{Test}\;\text{ response}\;\text{ time}-\text{Basal}\;\text{ response}\;\text{ time}}{\text{Cut‐off }\;\text{time}-\text{Basal}\;\text{ response }\;\text{ time}}\times 100. \end{array}$$

Immediately after the baseline latency assessment, mice were injected s.c. every 30 min with a set of progressively increasing morphine doses, each of which doubled the concentration of the preceding one (e.g., 5, 10, 20, 40, 80, 160 and 320 mg kg^−1^). Tail-flick latencies were tested 30 min after each dose and the subsequent dose was injected immediately. The progressive dosing procedure continued until the mice did not move their tails within the 10 s cutoff time. To investigate trends in body weight changes during the administration of various drugs, a set of mice were divided into four groups—NS-NS group, normal saline (NS) (100 *μ*L, s.c.) followed by NS (100 *μ*L, i.p.); Mo-NS group, morphine (10 mg kg^−1^, s.c.) followed by NS (100 *μ*L, i.p.); Mo-25 Cur group, morphine (10 mg kg^−1^, s.c.) followed by low-dose curcumin (25 mg kg^−1^, i.p.) and Mo-400 Cur group, morphine (10 mg kg^−1^, s.c.) followed by high-dose curcumin (400 mg kg^−1^, i.p.), and were compared on Day 1 and Day 8.

### 2.4. Semi-Quantitative Cytokine/Chemokine Protein Array

Blood samples were collected via the mandibular artery from a new set of mice on Day 8 from the NS-NS,Mo-NS, Mo-25 and Mo-400 Cur groups. The time interval between the two treatments was 15 min. For the blood collection, each sample contained 200 *μ*L of blood and 10 *μ*L of anticoagulant. Thereafter, the sample was immediately centrifuged at 1300 g for 20 min at 4°C and the supernatant was stored at −80°C until analysis. Each protein array required 300 *μ*L for adequate detection, with equal volumetric contributions from each mouse within the group (e.g., 75 *μ*L from each mouse in a group of four or 60 *μ*L from each mouse in a group of five). Cytokine expression was detected by using the RayBio Mouse Cytokine Antibody Array C Series 1000, which combines the mouse cytokine antibody array 3 (consisting of 62 cytokines) and 4 (consisting of 34 cytokines) to detect the expression of 96 cytokine expression in one experiment. All procedures were performed according to the manufacturer's instructions (RayBiotech, Inc., USA) and the signal intensity was scanned and quantified by densitometry. Positive control signals were generated with biotin-conjugated immunoglobulin G (IgG) antibodies, which are used to identify the orientation of and compare the relative expression levels among the different membranes. Changes in intensity ratio were considered significant if they satisfied either one of the following conditions: (i) the changes doubled or decreased by half or (ii) the intensity ratio became undetectable or vice versa.

### 2.5. Statistical Analysis

All data were expressed as the mean ± standard error (SE). SigmaPlot 10.0 was used to plot the cumulative dose-response curve. We used a linear regression model in the Statistical Package for Social Sciences version 10 (SPSS 10.0) to predict the 50% of maximal analgesic dose (AD50) and to subsequently generate corresponding 95% confidence intervals (CI). Tail-flick latencies for the same time periods within groups were compared by one-way analysis of variance (ANOVA) tests and *post hoc* comparisons between groups were performed using Duncan's test. A paired *t*-test was used to detect the trend of tail-flick latency within the same group at different time points. *P*-values < .05 were considered statistically significant.

## 3. Results

### 3.1. Inhibition of Body Weight Gain by Chronic Morphine and/or High-Dose Curcumin Administration

**Figure 2:**
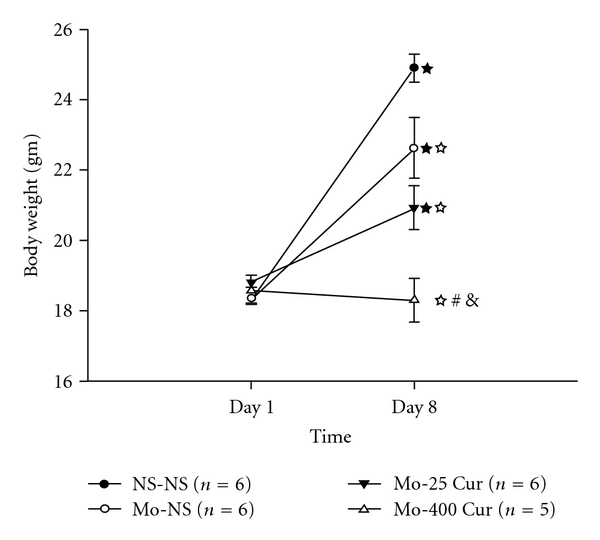
Effect of chronic morphine and/or curcumin administration on body weight after a 7-day injection. Formulas of injection on each day, including NS-NS, Mo-NS, Mo-25 Cur and Mo-400 Cur, represent normal saline (100 *μ*L, s.c.) followed by normal saline (100 *μ*L, i.p.), morphine (10 mg kg^−1^, s.c.) followed by normal saline (100 *μ*L, i.p.), morphine (10 mg kg^−1^, s.c.) followed by low-dose curcumin (25 mg kg^−1^, i.p.), and morphine (10 mg kg^−1^, s.c.) followed by high-dose curcumin (400 mg kg^−1^, i.p.) respectively. The interval between injections for each group was 15 min. ^★^ *P* < .001, = .005, = .021, different from their own baseline body weight on Day 1 in the NS-NS, Mo-NS and Mo-25 Cur groups, respectively. ^*☆*^ *P* < .05 compared with NS-NS group. ^#^ *P* < .05 compared with Mo-NS group. ^&^ *P* < .05 compared with Mo-25 Cur group.

We observed differences in body weight gain between the groups during the course of drug injection. As shown in Figure 2, body weight on Day 1 was not significantly different between the groups. Chronic administration of morphine (10 mg kg^−1^, s.c.) alone or in combination with low-dose curcumin (25 mg kg^−1^, i.p.) for 7 days resulted in an increased body weight on Day 8 when compared with each mouse's own baseline on Day 1. However, the observed body weight gain in the three study groups was still less than the gain seen in the control (NS-NS) group. No significant difference in weight gain was found between the morphine with low-dose curcumin (Mo-25 Cur) group and the morphine followed by NS (Mo-NS) group. However, chronic daily injection of high-dose curcumin after morphine administration further decreased the body weight gain compared with the morphine plus vehicle (Mo-NS) group.

### 3.2. Enhancement of Morphine's Antinociception by Acute High-dose Curcumin Injection

**Figure 3:**
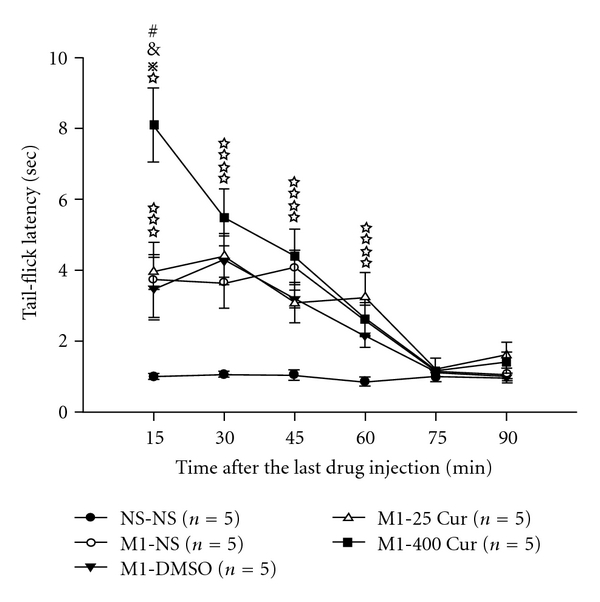
Acute tail-flick responses to drug combinations. Formulas of injection, including NS-NS, M1-NS, M1-DMSO, M1-25 Cur and M1-400 Cur, represent normal saline (100 *μ*L, s.c.) followed by normal saline (100 *μ*L, i.p.), morphine (1 mg kg^−1^, s.c.) followed by normal saline (100 *μ*L, i.p.), morphine (1 mg kg^−1^, s.c.) followed by DMSO (100 *μ*L, i.p.), morphine (1 mg kg^−1^, s.c.) followed by low-dose curcumin (25 mg kg^−1^, i.p.) and morphine (1 mg kg^−1^, s.c.) followed by high-dose curcumin (400 mg kg^−1^, i.p.), respectively. The interval between injections for each group was 15 min. ^#^ *P* < .05 compared with M1-NS group. ^&^ *P* < .05 compared with M1-DMSO group. ^*⋇*^ *P* < .05 < compared with M1-25 Cur group. ^*☆*^ *P* < .05 compared with NS-NS group.

To see if the acute injection of DMSO or curcumin could enhance morphine's antinociception, we first examined if there was an intrinsic antinociceptive effect exerted by DMSO (100 *μ*L, i.p.), low-, or high-dose curcumin. These drugs were injected i.p. 15 min after normal saline injection (100 *μ*L, s.c.), and we observed no antinociceptive effect in comparison with the NS-NS control group (data not shown). Second, a submaximal dose of morphine (1 mg kg^−1^, s.c.) was injected, followed by DMSO, and a low- or high-dose curcumin injection 15 min later, to examine if these agents could enhance morphine's antinociception (Figure 3). Tail-flick latency testing applied 15 min after the administration of the last drug demonstrated that all four drug combinations (morphine (1 mg kg^−1^, s.c.) followed 15 min later by normal saline (M1-NS), DMSO (M1-DMSO) and a low-dose (M1-25 Cur) or high-dose curcumin (M1-400 Cur)) yielded a significant antinociceptive effect when compared with the control (NS-NS) group within 15−60 min of the morphine injection, and this effect declined thereafter. Noticeably, high-dose curcumin enhanced morphine's antinociceptive capabilities when compared with other treatments; however, the enhancement only occurred 15 min after the last drug injection in M1-400 Cur group (Figure 3) .

### 3.3. Curcumin on the Development of Morphine Tolerance

**Figure 4:**
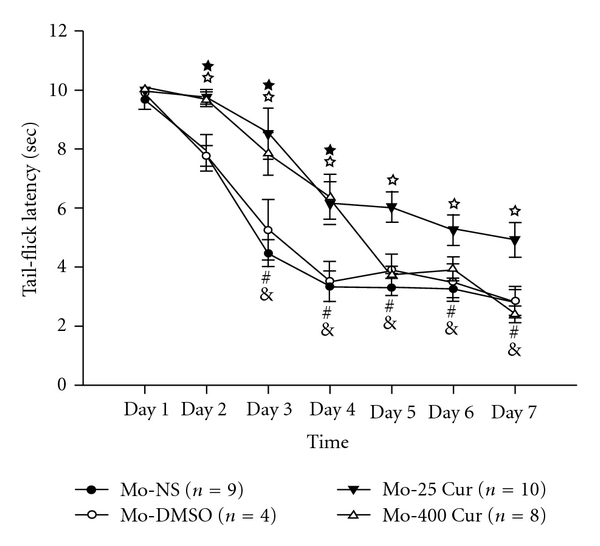
Tail-flick responses to chronic drug combinations for 7 days. Formulas of drug injections, including Mo-NS, Mo-DMSO, Mo-25 Cur and Mo-400 Cur, represent morphine (10 mg kg^−1^, s.c.) followed by normal saline (100 *μ*L, i.p.), morphine (10 mg kg^−1^, s.c.) followed by DMSO (100 *μ*L, i.p.), morphine (10 mg kg^−1^, s.c.) followed by low-dose curcumin (25 mg kg^−1^, i.p.) and high-dose curcumin (400 mg kg^−1^, i.p.), respectively, on each day. The interval between injections in each group was 15 min. Within the Mo-NS group, ^#^ *P* = .005 and ^&^ *P* < .001 versus Day 1. For Mo-25 Cur group, ^*☆*^ *P* < .05 compared with Mo-NS at each time; whereas for Mo-400 Cur group, ^★^ *P* < .05 compared with Mo-NS at each time.

Figure 4 demonstrates that administration of morphine s.c. at a dose of 10 mg kg^−1^ per day resulted in morphine tolerance on Day 2, and this tolerance was increasingly apparent in the following days. Low-dose curcumin attenuated the development of morphine tolerance from days 2 to 7. Conversely, high-dose curcumin only retained morphine's antinociception from days 2 to 4, with the effect diminishing from days 5 to 7.

### 3.4. Low-dose Curcumin Attenuated but High-dose Curcumin Worsened Morphine Tolerance after 7 Days Morphine Co-injection

**Figure 5:**
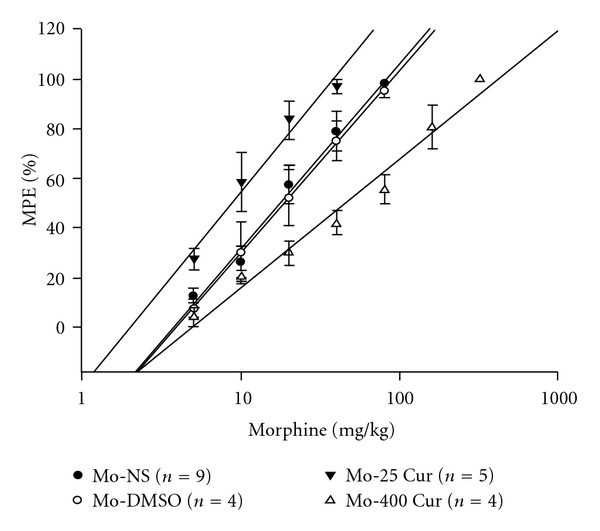
Cumulative dose-response curve on Day 8. Formulas of drug injections, including Mo-NS, Mo-DMSO, Mo-25 Cur andMo-400 Cur, indicate morphine (10 mg kg^−1^, s.c.) followed by normal saline (100 *μ*L, i.p.), morphine (10 mg kg^−1^, s.c.) followed by DMSO (100 *μ*L, i.p.), morphine (10 mg kg^−1^, s.c.) followed by low-dose curcumin (25 mg kg^−1^, i.p.) and high-dose curcumin (400 mg kg^−1^, i.p.), respectively, for 7 days. The interval between injections in each group was 15 min.

On Day 8, we plotted cumulative dose−response curves (Figure 5) and AD50 values for morphine were determined as previously described (Table 1). The AD50 value for morphine was 28 mg kg^−1^ in morphine-tolerant mice. The 95% CI for the AD50 value of morphine-tolerant mice with normal saline or DMSO injections overlapped, resulting in an insignificant shift of the dose-response curve. Chronic daily morphine injection followed by low-dose curcumin enhanced the antinociceptive effect of morphine in tolerant mice, with an AD50 of 13.0 mg kg^−1^ and a 2.15-fold shift in the dose-response curve. On the other hand, chronic daily morphine injection followed by high-dose curcumin significantly worsened morphine tolerance, with an AD50 of 98.1 mg kg^−1^ and a 3.5-fold shift in the dose-response curve.

### 3.5. Low- and High-dose Curcumin on the Expression of Cytokines/chemokines in the Development of Morphine Tolerance in Mice

In order to investigate the role of cytokines/chemokines in the development of morphine tolerance in mice, both with and without curcumin injection, up to 96 cytokines/chemokines were examined on Day 8 after injections for seven consecutive days. Even though no result reached a 2-fold increase or decrease against controls, 14 cytokines/chemokines appeared or disappeared after intervention and were therefore viewed as significant. When compared with the NS-NS group, s.c. morphine injection for 7 days eliminated the expression of fms-like tyrosine kinase 3 ligand (Flt3-ligand), macrophage-derived cytokine (MDC) and vascular endothelial growth factor (VEGF), whereas chronic daily morphine injection followed by low-dose curcumin restored the expressions of three proteins (Table 2). Compared with the Mo-NS group, injection of morphine with high-dose curcumin further eliminated the expression of a few more proteins, including leptin, VEGF receptor 1 (VEGFR-1), stem cell factor (SCF), regulated on activation, normal T expressed and secreted (RANTES), macrophage inflammatory protein-1*α* (MIP-1*α*), macrophage inflammatory protein-3*α* (MIP-3*α*), interleukin-13 (IL-13), cytokine-response gene 2 (CRG-2), soluble tumor necrosis factor receptor type II (sTNFRII) and tumor necrosis factor *α* (TNF*α*). In both low- and high-dose conditions, eotaxin was expressed in response to curcumin injection.

## 4. Discussion

**Figure 6:**
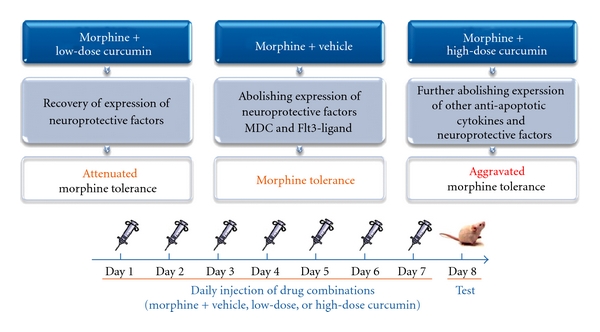
Biphasic effect of curcumin on morphine tolerance. After a 7-day drug intervention, AD_50_ values calculated from the cumulative dose-response curve on Day 8 showed that low-dose curcumin attenuates morphine tolerance but high-dose curcumin aggravates it.

In this study, we used a mouse model of morphine tolerance to measure body weight change, tail-flick latency and serum cytokine/chemokine expression. Morphine tolerance reduced body weight gain. For high-dose curcumin injection (Mo-400 Cur group in Figure 2), the body weight gain was reduced even further. Although previous studies reported a possible confounding effect of DMSO when used as a solvent [15], our results revealed that, at a concentration of 70%, neither acute (Figure 3) nor chronic (Figures 4 and 5, and Table 1) DMSO administration altered morphine antinociception. The main results are illustrated in Figure 6. Although acute low-dose curcumin did not enhance morphine's antinociceptive action (Figure 3), it did attenuate morphine tolerance during the treatment period (Figure 4). Acute morphine injection followed by high-dose curcumin 15 min later enhanced the antinociceptive effect of morphine when measured 15 min after the injection of the last drug (Figure 3). The effect of chronic daily high-dose curcumin injections, irrespective of whether it enhanced or preserved morphine's antinociception, was completely eliminated on days 5–7 (Figure 4). Morphine administration followed by low-dose curcumin produced a *∼*2-fold (28 mg kg^−1^ divided by 13 mg kg^−1^) increase in morphine-induced antinociception, whereas morphine administration followed by high-dose curcumin decreased morphine-induced antinociception by *∼*3.5-fold (98.1 mg kg^−1^ divided by 28 mg kg^−1^; Table 1 and Figure 5). As for the expression of cytokine/chemokine, no more than 2-fold changes were found. Therefore, we will only discuss those cytokines/chemokines for which absolute inhibition/zero expression was found in at least one group (Table 2).

On Day 8, morphine-tolerant mice were underweight when compared with control animals (Mo-NS versus NS-NS in Figure 2)—which was in agreement with a previous report [17], where the weight lag might be due to decreased eating and drinking behaviors, resulting from morphine withdrawal symptoms in the periods between injections. While a chronic daily morphine injection followed by low-dose curcumin did not further significantly reduce body weight in morphine-tolerant mice, the reduction in body weight due to high-dose curcumin treatment (Mo-400 Cur versus Mo-NS in Figure 2) corresponds with the inhibition of leptin expression observed in these mice (Table 2). A previous report also demonstrated that dietary curcumin could significantly reduce plasma leptin concentration, although in that study body weight and food intake are not altered [18]. Even though the leptin level correlates closely with body weight maintenance [19], it is also an important measure of body fat mass. Decreased leptin levels have been shown to cause obesity [20]; however, it also reflects a decrease in total body fat mass [19]. The latter effect could reasonably explain our observation since only a 7-day cycle of high-dose curcumin injection after morphine was able to significantly reduce body weight gain compared with morphine injection alone (Figure 2) and thereby eliminate the expression of leptin (Table 2).

Dietary supplements with similar high-dose curcumin (500 mg kg^−1^ per diet) decreased body fat and body weight gain in high-fat diet-fed mice by exerting an anti-angiogenic effect in the subcutaneous adipose tissue, in which the expression of VEGF and its receptor VEGFR-2 were down-regulated [21]. We screened the expression of three VEGF receptors, and the expression of VEGFR-1, but not VEGFR-2 or VEGFR-3 (data not shown), was eliminated by high-dose curcumin (Table 2). The discrepancy between the types of VEGF receptors involved might be attributable to the diet formula. While blockage of VEGFR-2 can limit fatty tissue expansion in high-fat diet-fed mice [22], inhibition of the VEGFR-1 signaling pathway can limit the adipose tissue in both mice fed with a high-fat diet and standard-fat diet [23]. Therefore, the reduced leptin level reflected the lower total fat mass in mice that were fed with a standard-fat diet and this may be associated with an inactivated VEGF-VEGFR1 pathway during the course of the experiment.

Expression of VEGF enhances the recruitment of endothelial progenitor cells [24] and is associated with distant metastases and poor tumor outcomes [25]. Although morphine has been reported to inhibit VEGF expression in myocardial ischemia [26] and chronic morphine administration (10 mg kg^−1^, s.c.) for 6 days can provide relief from cancer pain and inhibit tumor growth and metastasis in a mouse model [27], so far no direct link between VEGF and morphine tolerance has ever been reported. Our results showed that, after a consecutive 7-day morphine treatment (10 mg kg^−1^, s.c.), VEGF expression was completely eliminated in tolerant mice (Table 2). This provides an explanation, at least in part, for why chronic morphine treatments can reduce tumor growth and metastasis.

Proinflammatory cytokines and chemokines play an important part in the development of morphine tolerance [2, 3]. It is, therefore, not surprising that most of the cytokines and chemokines in the morphine plus vehicle group were comparable to the vehicle only group on Day 8 (Table 2) since, in morphine-tolerant animals, elevation of these proteins only appears 2 h after morphine administration and disappears 24 h later [3]. Although morphine tolerance shares a common neuroplastic pathway with neuropathic pain, it tends to exhibit more transitory biochemical alterations in response to a morphine challenge [9]. A similar presentation can be found in the patterns of elevated excitatory amino acid (EAA) levels in morphine-tolerant rats. Increases in cerebrospinal fluid (CSF)-EAAs, which are partially responsible for *N*-methyl-D-aspartate receptor (NMDAR) activation and therefore for the mechanism of chronic opioid-induced neuronal adaptation, are not present during the development of morphine tolerance [28–30] but are observed after morphine challenge or naloxone-precipitated morphine withdrawal [31]. Conceivably, our data suggest that, in the state of morphine tolerance, the expression of most cytokines/chemokines does not significantly change and instead is maintained in another paraphysiological balanced state.

In addition to VEGF, there are two other cytokines, MDC and Flt3-ligand, which were inhibited in morphine-tolerant mice, recovered by low-dose curcumin and secondarily inhibited by high-dose curcumin (Table 2). Neuronal apoptosis induced by prolonged exposure to morphine is associated with morphine tolerance [32], indicating that morphine could be a neurotoxin. MDC inhibits the neuronal apoptosis induced by the neurotoxin gp120 [33]; thus, the absence of MDC expression on Day 8 in the morphine-vehicle group (Table 2) might be related to morphine tolerance. Stimulation with Flt3-ligand is associated with antiapoptosis through the phosphorylation of the proapoptotic protein Bad [34]. Alternatively, Flt3-ligand can act synergistically with the nerve growth factor, which is essential for the survival of some sensory neurons, and thereby amplify its neurotrophic activity and increase neuronal survival [35]. Therefore, the elimination of Flt3-ligand expression might also be related to the development of morphine tolerance in the morphine-vehicle group. In our study, the chronic daily injection of low-dose curcumin after morphine reversed the inhibitive effect of chronic morphine on MDC and Flt3-ligand, an effect that might be at least partly responsible for the attenuation of morphine tolerance. However, these two proteins were again suppressed by high-dose curcumin. Our results demonstrate that the chronic daily injection of high-dose curcumin after morphine not only failed to attenuate morphine tolerance but it further worsened the condition.

Meanwhile, using protein array analysis, we found that many other neuroprotective cytokines/chemokines are inhibited by high-dose curcumin. SCF, which is strongly expressed in both the developing and adult CNS, protects neurons *in vivo* against apoptosis after spinal cord injury [36], and *in vitro* against camptothecin-induced apoptosis and glutamate excitotoxicity [37], which is one of the mechanisms that cause neuropathic pain and morphine tolerance [8]. There is evidence supporting the existence of a *β*-chemokine mechanism, that acts through MIP-1*α* and RANTES to contribute to neuroprotection against the neurotoxin gp120 [38]. MIP-3*α*, another constitutively expressed *β*-chemokine, was also shown to be suppressed in our study by co-injection of high-dose curcumin, although no protective effects of this chemokine against neuronal death have ever been reported. IL-13 has been shown to protect synoviocytes [39], normal airway epithelial cells [40] and ischemic hepatocytes [41] from apoptosis. Within the CNS, IL-13 is expressed exclusively by microglia cells, whose inflammation-induced activation can worsen CNS damage. The mRNA level of Toll-like receptor 4 (TLR4) was also found to have increased after intrathecal morphine administration for 7 days *in vivo* [2]—an effect which demonstrates a significant elevation of glial activation since only glia express TLR4 [42]. IL-13 may also control brain inflammation by inducing the death of activated microglia *in vivo*, resulting in the enhancement of neuronal survival [43]. Therefore, inhibition of IL-13 secretion by the chronic daily injection of high-dose curcumin after morphine may imply that the activated microglia have induced an over-inflammatory state in the CNS as compared with the morphine-vehicle group. As with IL-13, glia in the CNS have also been reported to be one of the principal sites of CRG-2 production in response to i.p. infection with mouse adenovirus-type 1 [44]. We speculate that CRG-2 functions in a way similar to IL-13, and therefore, reducing CRG-2 levels may have the potential for maintaining CNS glial cells, despite the fact that its corresponding pro-apoptotic abilities have only been reported in murine corneas with herpetic stromal keratitis [45].

Previous reports demonstrated that increased TNF*α* expression in the rat spinal cord is induced immediately after a 5-day infusion of morphine and that the neuroimmune response is prevented when morphine tolerance is attenuated by amitriptyline [30]. However, another time-course study revealed that the increased expression of TNF*α* mRNA in the dorsal horn of the lumbar spinal cord is observed only 2 h, but not 24 h, after a chronic 5-day infusion of morphine [3]. Although we did not observe any changes in TNF*α* expression 24 h after consecutive morphine injections (Table 2), it did participate in morphine tolerance in response to morphine. TNF*α* acts by binding to its two receptors, TNFR-I and TNFR-II, with the binding affinity for the latter several-fold greater than for the former [46]. The cleavage products of sTNFR-II, the soluble receptor form of TNFR-II, have a high affinity for TNF*α* and therefore retains its ability to function as a decoy binding site for this cytokine [47, 48]. sTNFR-II has been used to treat TNF*α*-mediated neuroinflammation [49] since it can antagonize the activity of TNF*α* by sequestering the cytokine away from the cell surface, and therefore, it can possibly lower TNF*α* response to morphine. In our results, chronic co-injection of high-dose curcumin with morphine abolished the expression of sTNFR-II (Table 2), which could have contributed to enhanced neuroinflammation in response to morphine injection and thereby worsened morphine tolerance.

Although morphine is immunosuppressive [50], the effects of sub-acute or chronic administration of morphine on immune function is limited, and most immune suppression parameters are observed after drug withdrawal [51]. As shown in our results, most proinflammatory cytokines, such as IL-1, IL-6 (data not shown) and TNF*α* did not significantly change 24 h after a 7-day morphine-vehicle administration or a low-dose curcumin treatment after morphine administration, as compared with the vehicle-only control. However, TNF*α* expression was inhibited by a daily injection of high-dose curcumin after morphine for 7 days, which could be explained by the direct dose-dependent effect of curcumin [6] or the existence of morphine withdrawal [52] precipitated by high-dose curcumin. Tolerance to morphine is often regarded as the gateway to the development of physical dependence, but these two phenomena are certainly dissociable and the underlying biochemical mechanisms may be different [53]. In this study, we did not record if morphine withdrawal developed on Day 8 and could not conclude if inhibition of TNF*α* was associated with morphine withdrawal. Eotaxin was neither constitutively expressed in the vehicle-only group nor in the morphine-vehicle group but it was induced in the curcumin-containing groups (Table 2). However, no difference in eotaxin activation was found between the low-dose and high-dose curcumin groups, and therefore, it is probably not related to the biphasic effect of curcumin on morphine tolerance.

For the past several years, curcumin has been viewed as a highly safe adjuvant for various conditions [4], and, before our study was conducted, it had no known dose-limiting toxicities [12]. The possibility of a biphasic behavior for curcumin was considered because it does not only induce cell death but also protects against it [54]. Although no clinical evidence was available, anecdotal evidence exists for its possible adverse effects, which included DNA damage-linked apoptotic cell death and temporal changes from an anti-oxidant to a pro-oxidant [5]. Although a previous report indicated that low to moderate doses of curcumin suppressed morphine analgesic tolerance in a dose-dependent manner, the tail-flick test was performed during a 90-min period after morphine injection [10]. However, in our investigation, the cumulative dose-response curve and AD50 values were calculated at 24 h after 7 days of consecutive morphine injections. It is possible that the extent of neuroinflammation became more obvious at 24 h than following morphine injection, necessitating further exploration. Our study is the first to demonstrate the adverse clinical effects of high-dose curcumin on morphine tolerance and the mechanism involved in the loss of expression of neuron-protective or antiapoptotic cytokines/chemokines, which may at least be partly responsible for the worsening of morphine tolerance.

Our study, however, has one limitation. Although the antibody-based protein array system offers many advantages over conventional enzyme-linked immunosorbent assays (ELISAs), including a higher sensitivity, greater range of detection and less variability [55], several alterations in the expression of cytokines were found in our protein array analysis. Therefore, it was difficult to determine which one of them is the key factor in worsening morphine tolerance. Pinpointing a specific cause was especially difficult because this kind of alteration, where we found absolute inhibition or zero expression, was not quantifiable even when repeated with a conventional ELISA and therefore could not be compared. Alternatively, it is possible that the cumulative effect of all the cytokine/chemokine changes is necessary to exacerbate morphine tolerance.

In conclusion, with this cytokine/chemokine protein array, we showed that while the expression of neuroprotective MDC and Flt3-ligand was eliminated in morphine-tolerant mice, chronic daily injections of low-dose curcumin after morphine administration recovered their expression and may be, at least in part, responsible for the attenuated morphine tolerance observed in these mice. Chronic daily injections of high-dose curcumin after morphine further abolished the expression of other antiapoptotic cytokines or neuroprotective factors and thereby worsened morphine tolerance. Curcumin by itself is a neuroprotectant [14, 56], but, as shown in our results, it might cause neurotoxicity when given chronically in high doses with morphine. In clinical practice, although curcumin is relatively safe to use as a single high dose orally [12], the effect of curcumin on morphine tolerance might be biphasic and therefore should be used cautiously.

## Figures and Tables

**Table 1:** Effects of DMSO and curcumin on the development of morphine tolerance.

Chronic treatment	AD_50_ (mg kg^−1^)	95% CI
Morphine-Normal saline	28.0	21.5–34.5
Morphine-DMSO	29.5	20.0–39.0
Morphine-25 mg kg^−1^ Curcumin	13.0	7.63–18.5
Morphine-400 mg kg^−1^ Curcumin	98.1	73.2–122

After 7 days of chronic treatment period, cumulative dose-response curves to acute morphine were generated on Day 8. AD_50_ were derived from these curves in Figure 5 CI represents confidence interval.

**Table 2:** Relative density of expression of serum cytokines/chemokines on Day 8 after a 7-day consecutive treatment.

Cytokine/chemokine	Treatment on each day			
	Normal saline (s.c.)-Normal saline (i.p.) (*n* = 5)	Morphine (s.c.)-Normal saline (i.p.) (*n* = 4)	Morphine (s.c.)-25 mg kg^−1^ Curcumin (i.p.) (*n* = 4)	Morphine (s.c.)-400 mg kg^−1^ Curcumin (i.p.) (*n* = 4)
Leptin	0.33	0.29	0.34	ND
VEGF	0.32	ND	0.30	ND
VEGF R1	0.35	0.33	0.33	ND
MDC	0.31	ND	0.29	ND
Flt3-ligand	0.30	ND	0.29	ND
SCF	0.29	0.27	0.29	ND
RANTES	0.30	0.27	0.31	ND
MIP-1*α*	0.41	0.31	0.43	ND
MIP-3*α*	0.38	0.30	0.40	ND
IL-13	0.31	0.28	0.32	ND
CRG-2	0.29	0.27	0.29	ND
sTNFRII	0.40	0.30	0.39	ND
TNF*α*	0.30	0.27	0.30	ND
Eotaxin	ND	ND	0.29	0.31

Each injection was delivered in 100 *μ*L vehicles and the interval between injections administered on the same day was 15 min. Abbreviations for specific cytokines/chemokines are as follows: VEGF for vascular endothelial growth factor, VEGF R1 for vascular endothelial growth factor receptor 1, MDC for macrophage-derived cytokine, Flt3-ligand for fms-like tyrosine kinase 3 ligand, SCF for stem cell factor, RANTES for Regulated on Activation, Normal T Expressed and Secreted, MIP-1*α* for macrophage inflammatory protein-1*α*, MIP-3*α* for macrophage inflammatory protein-3*α*, IL-13 for interleukin-13, CRG-2 for cytokine-response gene 2, sTNFRII for soluble tumour necrosis factor receptor type II, TNF*α* for tumor necrosis factor *α*. (ND: not detectable).
